# Profiles of Immune Infiltration and Prognostic Immunoscore in Lung Adenocarcinoma

**DOI:** 10.1155/2020/5858092

**Published:** 2020-06-08

**Authors:** Yanyan Li, Liping Tao, Weiyang Cai

**Affiliations:** ^1^Department of Ultrasound, The Second Affiliated Hospital and Yuying Children's Hospital of Wenzhou Medical University, Wenzhou, Zhejiang Province, China; ^2^Department of Gastroenterology, The First Affiliated Hospital of Wenzhou Medical University, Wenzhou, Zhejiang Province, China

## Abstract

Lung tissue is abundant with immune cells that form a powerful first defense against exotic particles and microbes. The malignant phenotype of lung adenocarcinoma (LUAD) is defined not only by intrinsic tumor cells but also by tumor-infiltrating immune cells (TIICs) recruited to the immune microenvironment. Understanding more about the immune microenvironment of LUAD could function in sorting out patients more likely with high risk and benefit from immunotherapy. Twenty-two types of TIICs were estimated based on large public LUAD cohorts from the TCGA and GEO datasets using the CIBERSORT algorithm. Then principal component analysis (PCA), meta-analysis, and single-sample gene set enrichment analysis (ssGSEA) were used to measure and evaluate the specific immune responses and relative mechanisms. Moreover, an immunoscore model based on the percent of immune cells was constructed via the univariate and multivariate Cox regression models, which provided an in-depth overview of the LUAD immune microenvironment and shed light on the immune regulatory mechanism. The differential expression genes (DEGs) were acquired based on the immunoscore model, and prognostic immune-related genes were further identified. GSEA and the protein–protein interaction network (PPI) further revealed that these genes were mostly enriched in many immune-related biological processes. It is hoped that this immune landscape could provide a more accurate understanding for LUAD development and tumor immune therapy.

## 1. Introduction

Lung adenocarcinoma (LUAD), a dominating subtype of non-small-cell lung cancer, is acknowledged and regarded as one of the most leading cause of cancer-related mortality worldwide, with a five-year survival rate of only 22.1% [1]. The primary reason accounting for this disappointing prognosis is that the majority of LUAD patients are diagnosed at advanced stage III or IV and the uneven efficiency of chemotherapy selection [2]. The admitted first-line treatment for NSCLC without driver mutations is cytotoxic chemotherapy, immune checkpoint inhibitors, or a combination of both modalities.

The lung surrounding contacting with with the outer world is abundant with immune cells that construct a powerful defense against noxious particles and microbes. Various immune cells, IFN response, immune checkpoints, HLA, cytokine, inflammation factor, and adoptive cell transplantation have been proven to make a great contribution in the progression of lung cancer. Immunotherapy, such as programmed cell death protein 1 (PD-1)/programmed death ligand 1 (PD-L1) checkpoint inhibitors and CTLA4, concentrates on revitalizing immunologic cells to release molecular components to defend against cancer cell in the tumor microenvironment. Two anti-PD-1 agents, pembrolizumab and nivolumab, and two anti-PD-L1 agents, atezolizumab and durvalumab, have been authorized by FDA for the treatment of lung tumor. It has been studied that pembrolizumab and nivolumab exhibited a surprising antitumor activity in advanced NSCLC with better overall survival (OS) and progression-free survival (PFS) than traditional second-line chemotherapy [3, 4]. Tumor PD-L1 expression is the only identified clinical biomarker to screen out patients most likely to respond to immunotherapy. Patients with high PD − L1 expression [TPS] ≥ 50% (tumor proportion score) and no EGFR or ALK genomic mutant are suitable for anti-PD-L1 agents. Disappointedly, the response rates for acceptable patients to PD-L1 have been variable. Only a proportion of high PD-L1 LUAD patients effectively respond to immunotherapy and gained a satisfactory clinical benefit [5], whereas some metastatic NSCLC patients given pembrolizumab were shown to have obvious longer PFS and OS regardless of PD-L1 expression [6]. More seriously, an increase of immunotherapy does take a leap of the probability of side effects without survival benefit in many cases. There are many reasons contributing to this phenomenon; anti-PD-L1 agents' therapeutic effects are limited by many biological characters, such as intratumoral heterogeneity and temporal change expression. At present, the clinical used flow cytometry and immunohistochemistry could not precisely meter the expression level of PD-L1, with low accuracy and credibility. In addition, some researches provided that there existed some intrinsic resistant mechanisms in the tumor immune environment for immunotherapy, such as improving the expression of immunosuppressive cells and checkpoint molecules such as tumor-associated macrophages (TAMs), T follicular helper (Tfh) and regulatory T cells (Tregs), and absence of antigenic proteins and antigenic presentation [7, 8]. The disorder of immune cells and molecules then generate the insensitivity of T cells, reprogram the phenotype of macrophage, alternate the traditional immune response signaling pathways, and engender T cell exclusion, which shut the door for the powerful immune therapy.

The ultimate aim is to identify distinguished LUAD patients that would benefit from immunotherapy, ensure optimal clinical response, minimize immune related adverse events, and decrease treatment costs. Therefore, it is warranted to propose other predictive immune therapy markers, not merely PD-L1, for the effectiveness of LUAD immunotherapy. Many ongoing clinical trials have been investigated focusing on the identification of predictive and prognostic biomarkers for immunotherapy. For example, peripheral blood inflammatory parameters have been shown to be correlated with poor prognosis and lower response rate to immune therapy in NSCLC [9, 10]; high tumor mutational burden level was associated with greater expression of neoantigens, which fosters anticancer immune response [10]; high LDH seems to negatively correlate with cytotoxic T lymphocyte activation and impairs aerobic glycolysis, which participated in the prediction of immune therapy [11]. However, the efficiency of these single indicators is still low and could not convince the public. They ignored the essence property of tumor immune microenvironment but concentrated on the index only reflecting - one side face of immunological indicators, impossibly whole. Thus, it is necessary to lucubrate the complexity of the LUAD microenvironment and identify subclasses of the tumor immune microenvironment existing in the LUAD tissues, which is beneficial for predicting and administering corresponding immunotherapeutics.

A comprehensive analysis of the tumor immune microenvironment in LUAD is imperatively required. In this study, we systematically described the constitutive pattern of the immune cell proportions and how it influenced progression of tumor development. Moreover, we constructed an immunoscore signature model to predict 1-, 3-, and 5-year overall survival for patients, with the hope to select patients for adjuvant immunotherapy and guide the development of new treatment options.

## 2. Materials and Methods

### 2.1. Data Acquisition

**Figure 1:**
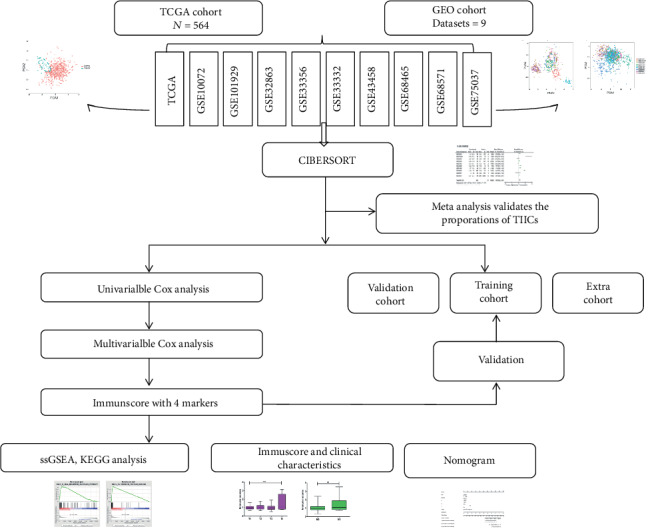
Study flowchart detailing the flow of sample collection and analysis.

This study utilized data from a public database. We retrospectively selected the LUAD gene expression and its clinicopathological data from the TCGA (https://cancergenome.nih.gov/) and GEO datasets (https://www.ncbi.nlm.nih.gov/geo/). We searched the key words “lung cancer” and “Homo sapiens”. All matrices with LUAD gene expression data (containing at least 20 samples) were considered eligible, with no specific exclusion criteria. All candidate series were assessed by two independent reviewers. A total of 371 normal and 990 LUAD cases were eligible for subsequent research. Raw microarray data Affymetrix were downloaded and normalized using the limma package. The platform profiles of the Affymetrix matrix were listed in table S1. The relevant clinical data from TCGA were retrieved and organized manually. The concrete working algorithm was demonstrated in Figure 1.

### 2.2. Inference of Infiltrating Immune Cells

Normalized gene expression data were used to calculate the proportions of 22 types of infiltrating immune cells via the CIBERSORT algorithm. CIBERSORT is a gene expression- based deconvolution algorithm, which infers cell type proportions in data from bulk tumor samples of mixed cell types using support vector regression [12]. CIBERSORT derives *P* values for the deconvolution for each sample using the Monte Carlo sampling, providing the confidence of the outcomes. At a threshold of *P* ≤ 0.050, the results of the inferred fractions of immune cell populations produced were considered precise and accurate [13]. Then, patients with CIBERSORT *P* ≤ 0.050 were considered eligible for the following analysis.

### 2.3. Systematic Meta-Analysis

Meta-analysis was referred as the standard method, offering an average impact estimate of the heterogeneity of effects across a series of results. In this study, we take advantage of the meta-analysis to layout the expression of particular immune cell infiltrate. Continuous outcomes were estimated as a standard mean difference (SMD) with a 95% confidence interval (CI). Setting *P* < 0.05 as the cut-off, we deeply explored the composition of TIICs in the LUAD to implement more convincing conclusions.

### 2.4. Evaluation of Tumor Immune Reaction Score

We obtained a series of genes in the immune-relative pathways from KEGG and published articles [13], then applied the single-sample gene set enrichment analysis (ssGSEA) to quantify the score by the GSVA and GSEABase packages [14]. The definition of each immune term was listed in the table S2. The correlation of the composition of the TIICs and the immune reaction were calculated by the Pearson correlation and showed by heat map.

### 2.5. Independence of the Prognostic Immunoscore for LUAD Survival Prediction

Only patients with CIBERSORT *P* ≤ 0.050 were selected for further survival analyses. Survival package was used to perform univariate Cox proportional hazard regression analysis to filter out prognostic immune cell, and then, multivariate Cox regression analysis was used to construct a powerful predictive immunoscore model. The optimal cut-off value was evaluated by the survminer package. With the median score established as the cut-off line, these LUAD patients were divided into the high- and low-immunoscore groups. The results of Cox regression analysis were showed by forest plot. A nomogram was generated via the survival, rms, and ggplot2 packages.

### 2.6. Random Grouping Method

The LUAD patients were randomly divided into the training and validation groups in a ratio of 6 : 4 using the stratified randomization method, which generated random values from a normal distribution with specified mean (0) and standard deviation (1) values and ordered them from high to low. These training and validation groups were both used to validate the precision of the immune model.

### 2.7. GSEA and PPI Analysis

We used the GSEA program to derive the enrichment scores of each immune-related term by calculated immunoscore [15]. Normalized gene expression data arranged by immunoscore were analyzed via the limma package to identify the immune DEGs, with *P* ≤ 0.05 and [logFC] ≥ 1.5 as the cut-off criterion. The connectivity degree of each node of the network was calculated by the STRING database and reconstructed via Cytoscape software.

### 2.8. Statistical Analysis

Continuous variables were exhibited for means, medians, range, and standard deviation (SD) and compared using an independent *t*-test or Wilcoxon test; categorical variables were compared between two groups by means of the Chi-squared test. Spearman' correlation coefficient was used for variable correlation. The associations of immune cell infiltrates and corresponding overall survival (OS) were analyzed by log-rank survival test, and the results were shown in the forest plot. To identify prognostic immune cells, the Cox proportional hazard regression model was employed. All statistical tests were two sided and *P* < 0.050 was considered statistically significant. Statistical analyses were conducted using R software and Stats.

## 3. Results

### 3.1. The Immune Landscape of the Microenvironment in LUAD

**Figure 2:**
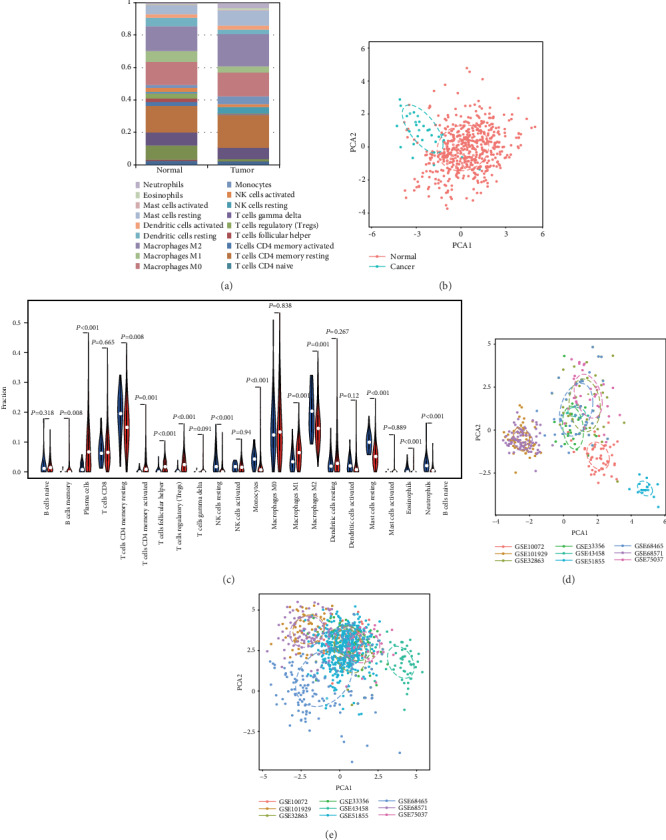
Landscape of microenvironment TIIC composition in LUAD. (a) The composition of TIICs of normal and RCC tissues. (b) The proportions of immune cells from normal and LUAD displayed distinct group bias clustering and individual differences in the TCGA dataset. (c) Violin plot of the proportions of the TIIC subpopulation (blue represents normal tissue, red represents LUAD). (d) PCA analysis of the subgroup of normal samples from the GEO dataset. (e) PCA analysis of the subgroup of LUAD samples from the GEO dataset.

After applying data filter criteria, 564 annotated lung adenocarcinoma samples with immune cell fraction were available for further analyses. We systematically described the immune microenvironment pattern of LUAD tissues from the TCGA cohort. As shown in Figures 2(a) and 2(b), the proportions of TIIC displayed distinct group bias clustering and expression pattern between normal and cancer tissues. The proportions of B cell memory, plasma, T cell CD4 memory activated, T cell follicular helper, T cell regulatory, and macrophage M1 were significantly improved, whereas the proportions of T cell CD4 memory resting, NK cell resting, monocytes, macrophage M2, and mast cell resting were downregulated (Figure 2(c)).

**Figure 3:**
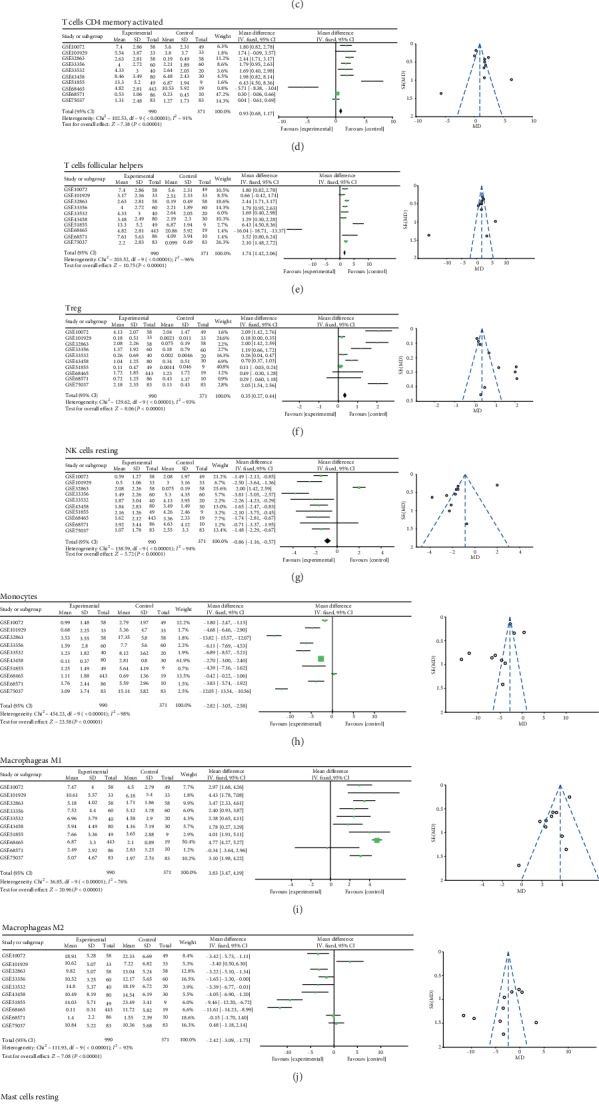
Meta-analysis verified each discrepant TIIC composition in LUAD.

To confirm the accuracy of the results of this study, the researchers inferred its accuracy in other independent LUAD datasets both containing rental tumor and adjacent normal specimens. Figure S1 summarized the compromise of TIIC subpopulations of each normal (Figure S1A) and LUAD tissues (Figure S1B) and CIBERSORT *P* value (Figure S1C). Although these LUAD cohorts were obtained from different platforms (Table S1) and variable signature matrix influences the accuracy of inferred TIIC constitutes, they did not show evident cohort bias both in normal and cancer tissues. We summarized each selected matrix and then eliminate the interaction between the components of the original data by PCA analysis (Figures 2(d) and 2(e), Figure S1). Then, precisely offering an average impact estimate of the heterogeneity of effects was performed to validate the proportions of each TIIC. Obviously, B cell memory (95% CI, 0.92-1.29; *P* < 0.01, *I*^2^ = 97%), plasma (95% CI, 3.58-4.67; *P* < 0.01, *I*^2^ = 91%), T cell CD4 memory activated (95% CI, 0.68-1.17; *P* < 0.01, *I*^2^ = 91%), T cell follicular helpers (95% CI, 1.42-2.06; *P* < 0.01, *I*^2^ = 96%), Treg (95% CI, 0.27-0.44; *P* < 0.01, *I*^2^ = 93%), and macrophage M1 (95% CI, 3.47-4.19; *P* < 0.01, *I*^2^ = 76%) exhibited a decreasing tendency, whereas T cell CD4 memory resting (95% CI, -2.75–-1.49; *P* < 0.01, *I*^2^ = 92%), NK cell resting (95% CI, -1.16–-0.57; *P* < 0.01, *I*^2^ = 94%), monocytes (95% CI, -3.05–-2.58; *P* < 0.01, *I*^2^ = 98%), macrophage M2 (95% CI, -3.09–-1.75; *P* < 0.01, *I*^2^ = 92%), Mast cell resting (95% CI, -4.11–-3.71; *P* < 0.01, *I*^2^ = 100%), and eosinophils (95% CI, -0.21–-0.06; *P* < 0.01, *I*^2^ = 94%) exhibited an increasing tendency (Figure 3). Together, we provided an in-depth overview of the LUAD TIIC subpopulation, which was tightly bounded with LUAD development and immune therapy.

### 3.2. TIIC Subpopulation Closely Correlated with Immune Signatures in LUAD

**Figure 4:**
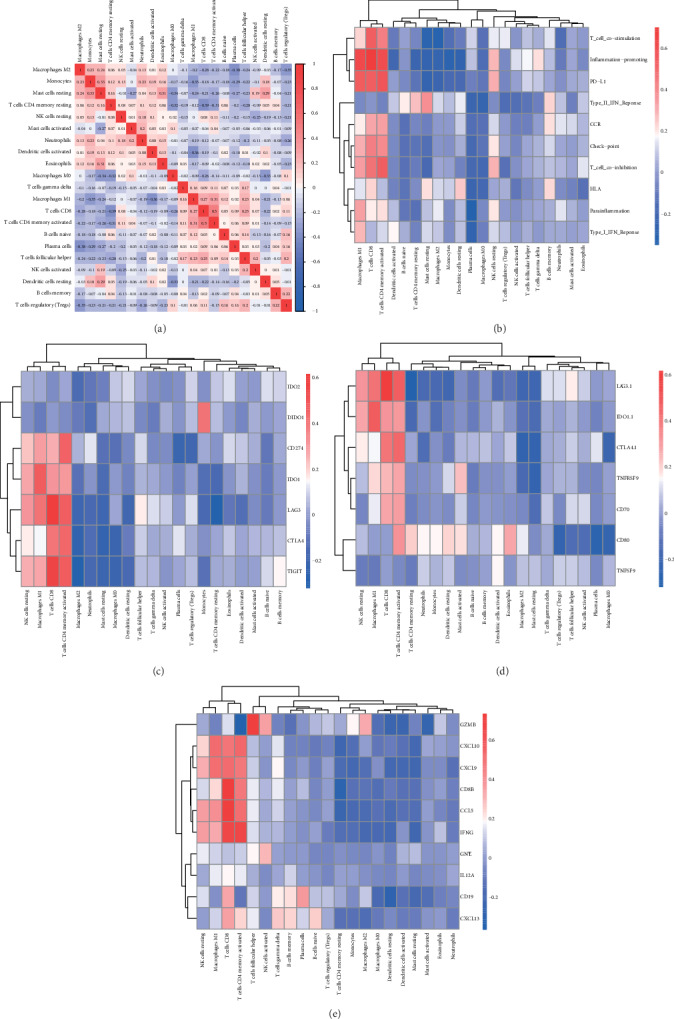
TIIC subpopulation was closely correlated with immune signatures. (a) The Spearman correlation matrix of all 22 immune cell proportions in the TCGA cohort. (b) The Spearman correlation between the proportions of infiltrating immune cells and specific immune signatures. (c) Correlation matrix of all 22 immune cell proportions and checkpoint response; (d). Correlation matrix of all 22 immune cell proportions and PD-L1 activity. (e) Correlation matrix of all 22 immune cell proportions and inflammation-promoting mechanisms.

To explore the correlation between various tumor immunity cell activities, we found that different TIIC subpopulations were weakly to moderately correlated, specially for T cell CD4 memory activated and T cell CD8 (Figure 4(a)). Next, we obtained a set of genes of the relative immune system from KEGG and published articles, and then used ssGSEA to quantify important immune signatures, including HLA expression, T cell cosimulation, inflammation-promoting mechanism, PD-L1 reaction, type II IFN response, type I IFN response, check point reaction, T cell coinhibition, parainflammation, and CCR. As shown in Figure 4(b), plasma, macrophage M0, and macrophage M2 were negatively correlated with immune signatures, whether macrophage M1 and T cell CD8 in mice. Moreover, we deeply explored the concrete association between TIIC checkpoint response (Figure 4(c)), PD-L1 reaction (Figure 4(d)), and inflammation-promoting mechanism(Figure 4(e)).

### 3.3. Establishment of Immunoscore for LUAD Patients

**Figure 5:**
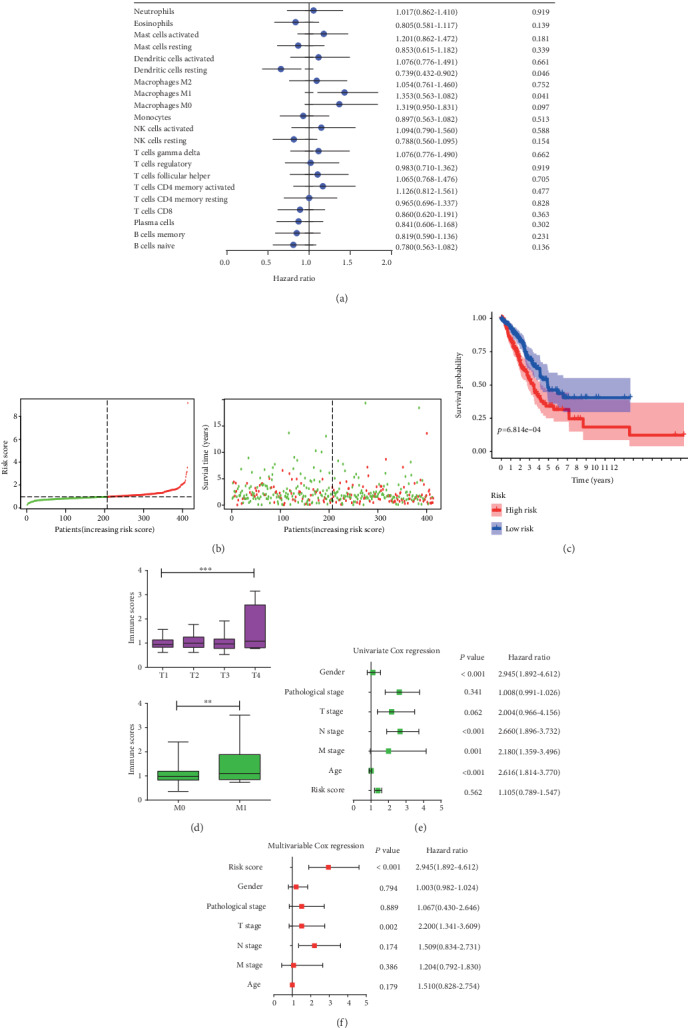
Construction of the immunoscore model. (a). Forest plots showed the association between each immune cell subsets and overall survival in the total TCGA dataset. Unadjusted hazard ratios are shown with 95 percent confidence intervals. (b). The distributions of the immune score and survival status for each LUAD patients. (c). Kaplan-Meier curves for the high- and low-immune score subgroup. (d) Association between the TIICs and clinicopathological features. Infiltrating immune cell function in distinguishing T and M stages. (e) Forest plot summary of the univariable analyses of overall survival of the immune score model. (f). Forest plot summary of the multivariable analyses of overall survival of the immune score model.

Considering the important role of the composition of the TIICs in the prognosis, we further explored their clinical significance. The unadjusted HRs and 95% confidence intervals for the median proportion of TIIC subsets were depicted in Figure 5(a). Macrophage M1 and dendritic cell resting were significantly associated with LUAD survival. Then, to identify a more predictive model with the best explanatory and informative efficacy, 4 subgroup immune cells were further selected to build an immunoscore model. We yielded the immune score for each tumor sample based on its immune proportion profiles: Formula = (the percentage of NK cell activated × 0.066) + (the percentage of macrophage M1 × 0.035) − (the percentage of dendritic cell resting × 0.028) + (the percentage of dendritic cells activated × 0.088). As shown in Figure 5(b), patients were divided into a high-immune and a low-immune group based on the median immunoscore. The overall survival of patients with a high-immune score was worse than that of those with a low-risk score (*P* = 0.814 − *e*04, Figure 5(c)). This immunoscore also had strong predictive power for T and M stages (Figure 5(d)).

To further investigate the prognostic value of the immune signature model, univariable and multivariable Cox regression analyses were performed considering immune score, sex, T stage, N stage, M stage, and pathological stage. The results of univariable and multivariable analyses of the above clinicopathological variables were presented in Figures 5(e) and 5(f). In conclusion, the immunoscore was a significantly independent prognostic factor for LUAD patients.

### 3.4. Variation in Prognostic Effect of Immunoscore in Intra- and Extracohort

To confirm that the proposed immunoscore model has a powerful prognostic value in different populations, the formula was applied to the validation cohort and also to the other cohort. We randomly divided total patients in the primary and validation cohort. Consistent with the findings in the total TCGA cohort, patients in the high-immunoscore group had a significantly lower overall survival rate than those in the low-immunoscore group in both the validation cohort (HR = 2.57, 95% CI: 1.537-4.301) and the training cohort (*HR* = 1.419, 95% CI: 1.152-1.844) (Table 1). In line with these findings, the immunoscore model was also validated to be independently associated with survival in extracohort GSE101929 and GSE6857 (Figure S2, only this two cohorts possessed clinical information in the above meta-analysis data).

### 3.5. Prognostic Nomograms for Prediction of LUAD Patients' Overall Survival

**Figure 6:**
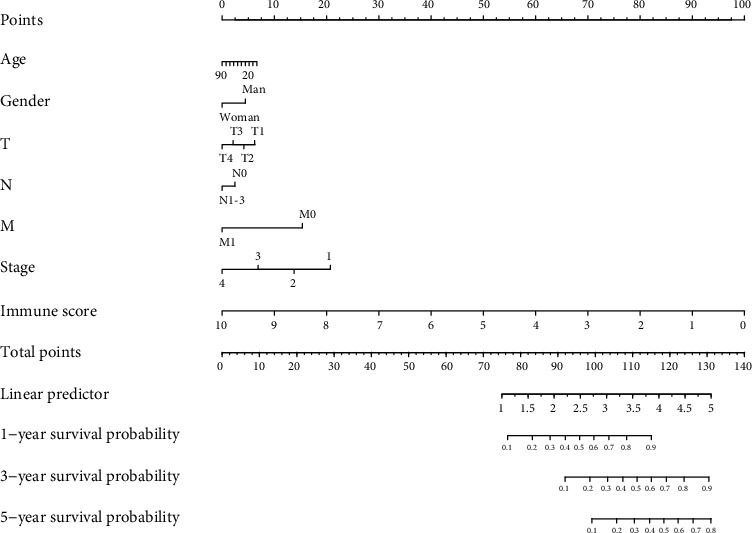
The nomogram to predict the 1-, 3-, and 5-year overall survival rates of LUAD patients.

To develop a practical method for clinicians to predict the LUAD survival probability, we constructed a prognostic nomogram that integrated the immunoscore, age, sex, T stage, N stage, M stage, and pathological stage. These variables from the Cox proportional analysis were all considered. As shown in Figure 6, the immunoscore contributed the most risk points (ranged 0 to 10), whereas the other clinical factors contributed much less (ranged 0 to 20). In general, the immunoscore was an independent risk predictor for the overall survival for LUAD patients.

### 3.6. Functional Analysis of Immune-Relative Genes

We compared the gene expression profiles between the high and low immunoscores and identified the KEGG pathways that were enriched in the cancerous immune activity by GSEA. It suggested that the high-immunoscore group was significantly enriched in B cell receptor signaling pathway, autoimmune disease, primary immunodeficiency, and T cell receptor signaling pathway (Figure S3). These results confirmed the elevated immune activity in the progression of LUAD.

**Figure 7:**
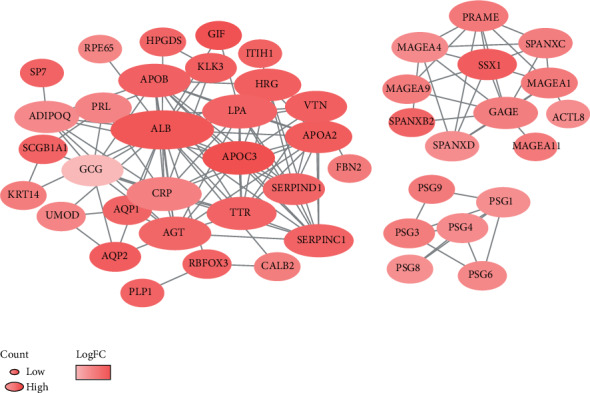
PPI network of immune prognostic genes. The color of a node in the PPI network reflects the log(FC) value of gene expression; the size of node indicated the number of interacting proteins with the designated protein.

To investigate the potentially altered molecular mechanisms, immune-relative genes were selected by comparing the high- and low-immunoscore groups. Totally, 24 genes were upregulated and 24 genes downregulated. The relationship and function of immune-relative genes were revealed using the PPI network, and there exist three significant immune relative modules (Figure 7). In the most significant one (left), 95 edges involving 30 nodes were formed in the network (Table S3). ALB, APDC3, LPA, APDC3, and CRP were hub genes and remarkable for having many connections with others.

## 4. Discussion

In many studies, the important impact of immune cells in determining the progression of the LUAD pathogenesis has been proven. However, owing to technical limitations, relative studies have only heavily relied on limited repertoire of immune phenotypic markers. The defects of single-agent immunotherapy are restricted to a small number of patients, with a response rate of 11-21% [6]. Immunotherapy concentrates on the activation of immunologic molecular components to defend against the cancer cell in the tumor microenvironment. Thus, it is important to deeply explore the influence of the tumor microenvironment on LUAD immunotherapy, which is the key to overcoming de novo resistance. Compared with traditional single-factor predictors, a comprehensive systematic assessment of the immune system is urgently needed to better understand the development of LUAD.

In our study, LUAD gene expression data from the TCGA database was analyzed based on CIBERSORT method, we found that the proportions of TIICs varied between the normal and cancer tissues. CD4 memory resting, macrophage M0, and macrophages M2 ranked the top three proportions of TIICs in LUAD. CD4 memory resting T cells can differentiate into a variety of helper T cells and regulatory T cells, which play an important role in adaptive immune response, participating in humoral immunity and regulation of cellular immunity [16]. Meanwhile, tumor-associated macrophages (TAM) have been proved to be the most important immune cells in the tumor stroma and played an important role in the occurrence and development of malignant tumors, accounting for more than 50% of the total number of immune cells in the tumor stroma [17, 18].

Further research showed that B cell memory, plasma cells, T cell CD4 memory activated, T cell follicular helper, Tregs, and macrophage M1 were significantly increased in LUAD compared with the adjacent normal tissues. In contrast, T cell CD4 memory resting, NK cell resting, monocyte, macrophage M2, mast cell resting, eosinophils, and neutrophils were decreased in LUAD compared with normal tissues. Then, the proportions of TIIC were validated in GEO the database, and the trend was consistent with our previous results. The varying degree of infiltration of some immune cells between the tumor and adjacent normal tissues suggested that these cells played an important role in the development of LUAD. Macrophages are the main immune-infiltrating cells in tumors, the key cell types that link inflammation and cancer [19]. The major states of macrophages, macrophage M1 and macrophage M2, differently infiltrated between the tumor and adjacent normal tissue. Macrophages have a series of continuous functional states, and M1-type and M2-type macrophages are the two extremes of this continuous state [20]. Macrophage M1 activates the production cytokine, recruited the proimmunostimulating leukocytes TME, which ultimately leads to the phagocytosis of tumor cells [21]. However, macrophage M2 was reported to have an opposite function in tumor; it promotes the development of tumor via the breakdown of basement membrane, recruitment of leukocytes recruitment, angiogenesis, and immune suppression [22, 23]. Interestingly, previous studies showed that the increased level of macrophage M1 level was associated with better prognosis of tumor [24, 25], while the increase of macrophage M2 indicates that the prognosis of tumor patients is poor [26]. Studies have demonstrated that cytotoxic activity in the environment of the tumor would be increased through reversion of macrophages from M2 to M1 phenotypes [27]. Thus, scientists proposed that reprogramming the macrophage from M2 to M1 phenotypes in different ways increases the antitumor activity of macrophages and provides new ideas for the treatment of tumors. Some studies have been carried out. For example, in a tail metastasis model established by breast cancer, the activation of NF-*κ*B in macrophages lead to a reversion from macrophage M2 to macrophages M1 and ended up with an increased apoptosis of tumor cells and a reduction of lung metastases [28]. In addition, more studies reported that plasma cells play a positive role in antitumor immunity, indicating a positive prognostic effect in human cancers [29]. The number of follicular T helper cells increased significantly in MLD patients, and the proportion of follicular T helper cells decreased in effective treatment patients, which could be used as an indicator of efficacy [30]. Among the 13 immune cells, other than those discussed above, some are poorly investigated in LUAD. As for their biological function in LAUD, we believe that further investigations are urgently needed.

The interaction of programmed cell death ligand-1 (PD-L1) interaction with programmed cell death protein-1 (PD-1) provides an important target for blockade-based immunotherapy in LUAD [3, 31, 32]. Thus, based on our researches, we would like to explore the association between TIICs and PD-L1 reaction was further explored. The results showed that the expression of PD-L1 was positively associated with macrophage M1, T cell CD8, and T cell CD4 memory activated. The association between macrophages and anti-PD-1 therapy has been reported before. Previous studies of anti-PD-1 therapy in osteosarcoma (OS) have shown that anti-PD-1 therapy can cause phenotypic metastasis of macrophages from M2 to M1, resulting in regression of OS pulmonary metastasis. Macrophage depletion significantly reduced the efficacy of anti-PD1, confirming their role in anti-PD-1 against OS pulmonary metastasis [33]. Another study showed that the expression of PD-L1 negatively correlates with phagocytic ability against tumor cells, and the blockade of PD-1-PD-L1 increased the phagocytosis ability of macrophage and reduced the progression of tumor, as well as prolonged survival time of mice in mouse models of cancer, indicating that anti-PD-1 therapy may function through a direct effect on macrophages [34]. In conclusion, the above results suggested that macrophage cells played a key role in the anti-PD-1 therapy of LUAD and can be a potential target of immunity therapy of LUAD in the future.

The result of univariate analysis showed that not overall immune cell infiltration but a certain TIIC subpopulation was related to the prognosis of LUAD. In this way, a Cox regression model was constructed based on the estimated fractions of signature immune cells. In recent years, several models based on immunoscore have been published to quantify the immune contexture and provide a statistically robust parameter for prognosis in patients with various types of solid tumor. Our study showed that patients with high immune scores have shorter survival time than patients with low immune scores, and this result was validated in a testing dataset and two independent databases from the GEO database. Further studies showed that the immune score was significantly related to the T stage and M stage. TNM staging system was limited in clinical practice, due to the ignorance of age, gender, or clinical stage of patients [35]. We believe our immunoscore signature is a great complement to TNM staging system. Thus, a nomogram based on age, gender, T stage, N stage, M stage, tumor stage, and immunoscore signature was constructed, with which we can accurately predict the prognosis of LUAD patients.

The potential mechanism for the differences in immune score was explored through GSEA. The results showed that a series of pathways associated with immune responses were significantly enriched in patients with high immune score. Moreover, DEGs of the high- and low-immune score group were identified and sent to PPI network. The results show that ALB, APOC3, LPA, AGT, and SSX1 were the hub gene of the module of immune-related cells. Totally, the above results suggested that immune responses play a critical role in the development, diagnosis, and treatment of LUAD and strong attention should be paid to it.

Despite the remarkable results of our research, we should also recognize the limitations. Firstly, our model was built based on the TCGA database and was validated in the GEO dataset, and multicenter clinical date should be collected to validate our model. Secondly, the level of tumor-associated immune cells was estimated using CIBERSORT method, and future studies should be needed to confirm the findings.

In summary, we provided an in-depth review of TIIC subsets in LUAD, which were closely related to the development of LUAD and immunotherapy. The immune score signature was constructed based on four survival-related immune markers. In addition, a nomogram was constructed by combining the characteristic immune score signature and meaningful clinical factor, providing an accurate model for predicting the prognosis of patients with LUAD, However, experimental researches on the mechanism between immune cells and LUAD are still needed in the future.

## Figures and Tables

**Table 1:** Validation of the immunoscore results in the training and validation cohort in the TCGA database.

Variable	Training cohort		Validation cohort	
	*P* value	Hazard ratio (95% CI)	*P* value	Hazard ratio(95% CI)
M	0.897		0.810	
M0	0.698	0.753 (0.180-3.149)	0.541	0.642 (0.155-2.663)
M1	0.647	0.723 (0.180-2.898)	0.622	0.699 (0.168-2.901)
N	0.621		0.759	
N0	0.505	0.617 (0.149-2.554)	0.766	0.710 (0.074-6.828)
N1-3	0.329	0.581 (0.196-1.727)	0.457	0.693 (0.264-1.822)
T	0.241		0.813	
T1	0.152	0.285 (0.051-1.586)	0.314	7.388 (0.203-14.299)
T2	0.459	0.590 (0.146-2.385)	0.806	0.842 (0.214-3.317)
T3	0.606	0.842 (0.438-1.620)	0.990	0.922 (0.266-3.700)
T4	0.800	1.724 (0.279-5.236)	0.796	1.208 (0.288-5.067)
Stage	0.144		0.785	
Stage1	0.133	0.408 (0.126-1.315)	0.326	0.520 (0.141-1.921)
Stage2	0.023	1.263 (1.016-1.832)	0.389	0.555 (0.145-2.119)
Stage3-4	0.418	0.802 (0.470-1.368)	0.711	0.877 (0.438-1.757)
Gender	0.648		0.782	0.682 (0.216-1.243)
Man	0.369	0.781 (0.455-1.340)	0.619	0.856 (0.463-1.582)
Woman	0.364	0.787 (0.469-10320)	0.546	1.207 (0.655-2.223)
Immune score	*0.029*	2.570(1.537-4.301)	0.001	1.419(1.152-1.844)
	0.062	1.016(0.969-1.021)	0.325	1.027(0.988-1.036)

## Data Availability

The data are available in the TCGA and GEO datasets.

## Supplementary Materials

Supplementary Materials Supplementary Figure 1: summary of GEO inferred GEO TIIC composition. (A) Summary of GEO chips concrete immune cell subset proportions of normal lung tissues. (B) Summary of GEO chips concrete immune cell subset proportions of LUAD tissues. (C) The proportion of samples with different *P* value threshold between studies. Supplementary Figure 2: validation of the immunoscore results in other cohorts from the GEO database. Kaplan-Meier survival curves showed that this immunoscore was associated with overall survival in GSE101929 and GSE68571. Supplementary Figure 3: GSEA differentiates the molecular mechanism between the high- and low-immune score group. Table S1: the detailed information of GEO chips. Table S2: the specific markers of immune signatures for ssGSEA. Table S3: the specific construction of PPI interaction.
