# Supplementary material for: Profiles of Immune Infiltration and Prognostic Immunoscore in Lung Adenocarcinoma
Source: Biomed Res Int. 2020 Jun 8;2020:5858092. doi: 10.1155/2020/5858092 (PMC7301220; doi:10.1155/2020/5858092)
Supplement: Supplementary Materials — Supplementary Figure 1: summary of GEO inferred GEO TIIC composition. (A) Summary of GEO chips concrete immune cell subset proportions of normal lung tissues. (B) Summary of GEO chips concrete immune cell subset proportions of LUAD tissues. (C) The proportion of samples with different P value threshold between studies. Supplementary Figure 2: validation of the immunoscore results in other cohorts from the GEO database. Kaplan-Meier survival curves showed that this immunoscore was associated with overall survival in GSE101929 and GSE68571. Supplementary Figure 3: GSEA differentiates the molecular mechanism between the high- and low-immune score group. Table S1: the detailed information of GEO chips. Table S2: the specific markers of immune signatures for ssGSEA. Table S3: the specific construction of PPI interaction. [file 5858092.f1.pdf]

Supplementary Figure1: Summary of GEO inferred GEO TIICs composition. A. Summary of GEO chips concrete immune cell subset proportions of normal lung tissues; B. Summary of GEO chips concrete immune cell subset proportions of LUAD tissues; C. The proportion of samples with different P- value threshold between studies.

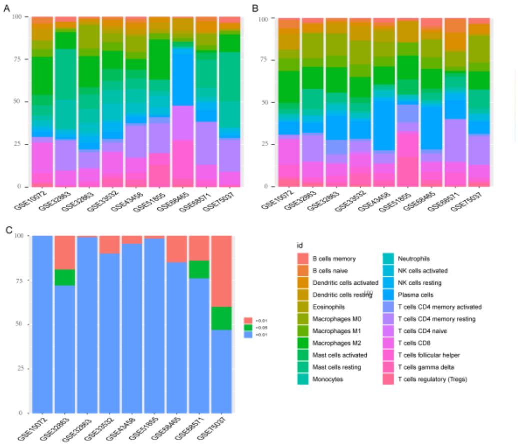

Supplementary Figure 2: Validation of the immunoscore results in other cohorts from the GEO database. Kaplan–Meier survival curves showed that this immunoscore were associated with overall survival in GSE101929 and GSE68571.

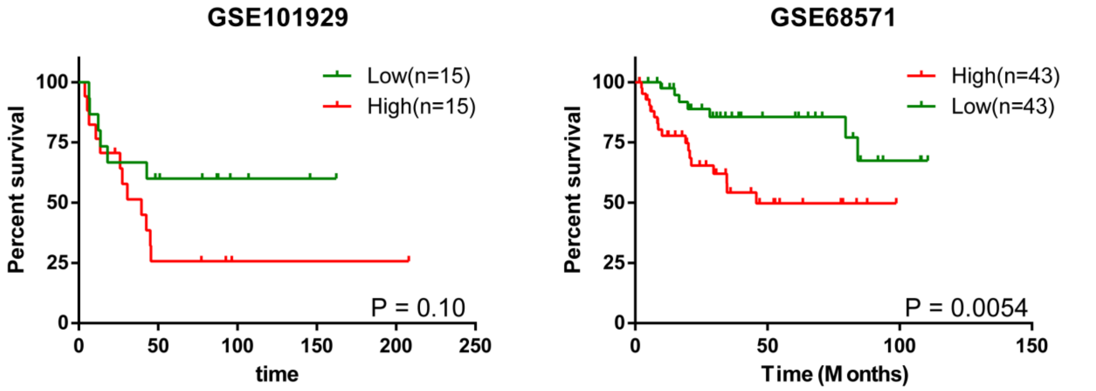

Supplementary Figure 3: GSEA differentiates the molecular mechanism between high - and low - immune score group;

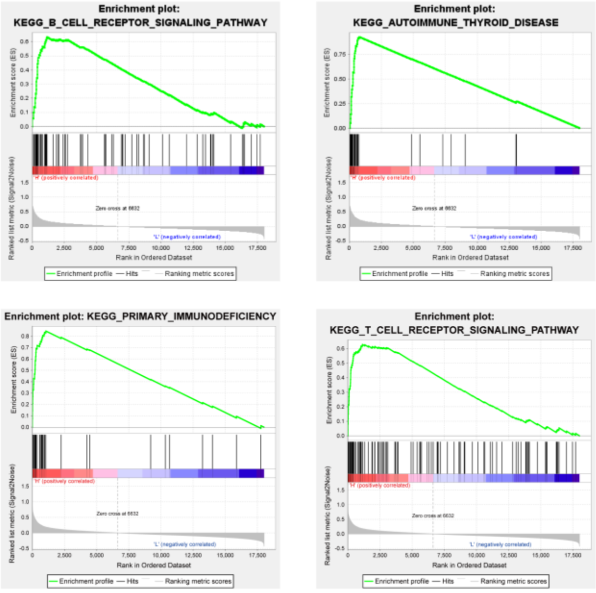

Table S1 The detailed information of GEO chips

**Accession**	**Platform**	**Number of normal samples**	**Number of cancer samples**
**GSE10072**	GPL80	49	58
**GSE101929**	GPL570	33	33
**GSE32863**	GPL6947	58	58
**GSE33356**	GPL96	604	60
**GSE33532**	GPL96	20	40
**GSE43458**	GPL570	30	80
**GSE51855**	GPL570	9	49
**GSE68465**	GPL15207	19	443
**GSE68571**	GPL6947	10	86
**GSE75037**	GPL6947	83	83
**Total**	1361	371	990

Table S2 The specific markers of immune signatures for ssGSEA

Immune response	Gene symbol
PD-L1 Response	IDO2, CD274, IDO1, CTLA4, LAG3, TIGIT,TNFSF9, CD80,CD70
Type I IFN Reponse	DDX4, IFIT1, IFIT3, IRF7, ISG20, MX1, MX2, RSAD2, TNFSF10
Type II IFN Reponse	GPR146, SELP, AHR
Check-point	IDO1, LAG3, TIM-3, IDO2, CD274/PD1, CTLA4, TIGIT
HLA	HLA-E, HLA-DPB2, HLA-C, HLA-J, HLA-DQB1, HLA-DQB2, HLA-DQA1, HLA-DQA2, HLA-A, HLA-DMA, HLA-DOB, HLA-DRB1, HLA-H, HLA-B, HLA-DRB5, HLA-DPB1, HLA-DRA, HLA-DRB6, HLA-L, HLA-F, HLA-G, HLA-DMB, HLA-DPA1
MHC class I	B2M, HLA-A, TAP1
Parainflammation	CXCL10, PLAT, CCND1, LGMN, PLAUR, AIM2, MMP7, ICAM1, MX2, CXCL9, ANXA1, TLR2, PLA2G2D, ITGA2, MX1, CD276, TIRAP, IL33, PTGES, TNFRSF12A, SCARB1, CD14, BLNK, IFIT3, RETNLB, IFIT2, ISG15, OAS2, REL, CD44, RRPAG, BST2, OAS1, NOX1, PLA2G2A, IFIT1, IFITM3, IL1RN
Inflammation-promoting	CCL5, CD19, CD8B, CXCL10, CXCL13, CXCL9, GNLY, GZMB, IFNG, IL12A, IL12B, IRF1, PRF1, STAR1,TBX21
T cell co−stimulation	CD2, CD226, CD27,CD28, CD40LG, ICOS, SLAMF1, TNFRSF18, TNFRSF25, TNFRSF4, TNFRSF8, TNFRSF9, TNFSF14
T cell co−inhibition	BTLA, C10orf54, CD160, CD244, CD274, CTLA4, HAVCR2, HAVCR2, LAG3, LAIR1, TIGIT

Table S3 The specific construction of PPI interaction

Gene	Count	Fold change
APOA2	10	2.233579
APOB	10	1.644275
LPA	12	1.438688
SERPINC1	13	1.310833
ALB	11	2.698981
ADIPOQ	24	-2.64953
GCG	9	-6.83411
PSG3	8	-1.42882
PSG4	4	-3.0064
CRP	4	-1.83787
GAGE1	13	-1.58838
RBFOX3	9	1.256252
UMOD	3	-2.23816
PSG1	5	-3.2024
PRAME	7	-0.96867
AGT	12	1.022967
SSX1	7	2.012611
TTR	11	1.273303
PRL	6	-2.2085
HRG	10	1.792114
ITIH1	1	1.480321
SP7	1	1.383522
APOC3	12	3.6009
KLK3	4	1.869852
SPANXC	5	-1.6484
VTN	8	1.818103
MAGEA4	6	-2.78121
CALB2	2	-1.5283
SPANXD	5	-3.13902
ACTL8	1	-3.05311
FBN2	1	-1.13477
MAGEA9	3	-1.37312
AQP1	3	1.2
SCGB1A1	3	1.273383
PLP1	1	1.368618
SPANXB2	2	1.235
SERPIND1	8	1.171029
PSG9	2	-1.19542
MAGEA1	6	-1.18092

AQP2	4	2.076496
PSG8	2	-3.30832
HPGDS	2	1.153901
PSG6	3	-2.53943
GIF	2	3.426055
KRT14	2	-1.41315
MAGEA11	1	-1.00638
RPE65	1	-2.4149
